# First molecular detection of small ruminant lentivirus in a roe deer (*Capreolus capreolus*) in Italy

**DOI:** 10.3389/fvets.2026.1900987

**Published:** 2026-10-09

**Authors:** Sara Mrabet, Paola Gobbi, Lucia Toso, Alice Ranucci, Luca Tassoni, Elena Tinelli, Giulia Costantino, Monica Giammarioli, Carmen Iscaro, Marco Gobbi, Francesco Feliziani, Maria Serena Beato

**Affiliations:** 1 National Reference Laboratory for Ruminant Retroviruses (CEREL), Istituto Zooprofilattico Sperimentale dell’Umbria e delle Marche “Togo Rosati” (IZSUM), Perugia, Italy; 2 Animal Health Diagnostic Department, Marche, Istituto Zooprofilattico Sperimentale dell’Umbria e delle Marche “Togo Rosati” (IZSUM), Tolentino, Macerata, Italy

**Keywords:** interspecies transmission, Italy, roe deer, small ruminant lentivirus, wildlife

## Abstract

Small Ruminant lentiviruses (SRLVs) infect sheep and goats worldwide, representing one of the most insidious viral infections in this species. Presence of antibodies against SRLVs was reported in wild ruminants in the USA and Europe and detection of virus was reported from wild ibex (*Capra ibex*) and red deer (*Cervus elaphus*) in Europe. This evidence suggests the potential role of wild ruminants as SRLVs reservoirs. A passive surveillance program was conducted in roe deer (*Capreolus capreolus*) from 2023 to 2025 in Central Italy, to investigate the presence and circulation of SRLVs. In 2025, one female roe deer was detected positive for SRLVs by PCR; sequencing and phylogenetic analysis indicated the virus belonged to the A23 sub-genotype, clustering with SRLVs previously identified in domestic species in Southern Italy. Even though this is an isolated case, this first-ever detection of SRLV in a roe deer suggests the possibility of spillover to/from domestic small ruminants and highlights the importance of long-term wildlife monitoring to clarify the extent and dynamics of SRLV circulation in wild cervid populations.

## 1 Introduction

Small Ruminant Lentiviruses (SRLVs) belong to the genus Lentivirus, family *Retroviridae*. They include Maedi-Visna Virus (MVV) and caprine arthritis-encephalitis virus (CAEV) (1). Initially, they were considered host specific. Nowadays, several studies have demonstrated that they are capable of cross- and co-infecting sheep and goats (2–4). The genome consists of two positive single-stranded RNA molecules of approximately 8–10 kb (5), which are transcribed into proviral DNA by the retrotranscriptase enzyme once the virus has infected the host cell. The genomic organization is similar to other Retroviruses. The genome is flanked by non-coding long terminal repeats (LTRs), which are essential for viral replication as sites of transcriptional initiation (6). The genome contains structural (*gag*, *pol* and *env* gene) (1, 5, 7) and regulatory genes (vif, rev and vpr-like) (8–10). Phylogenetic classification criteria of SRLVs were established by Shah et al. (11), defining four genotypes (A, B, C, D), based on the characterization of the *gag-pol* region and the *pol* gene. A fifth genotype, named E, was isolated in Italy in 2007 (12). SRLVs show a high genetic variability, with genotype A showing the greatest heterogeneity (from A1 to A27), followed by B genotype (from B1 to B5), and genotype E (sub-genotypes E1 and E2) (3, 13, 14). However, recent genetic characterization of SRLV circulating in Italy suggested that the current classification may lead to an excessive diversification not supported by robust genetic distances (15). SRLVs induce a lifelong persistent infection that may result in asymptomatic animal shedders (6) and in different clinical manifestations, from a multisystemic form to localized ones (pulmonary, mammary, nervous, joints) (5), depending on viral tropism, infected species and/or breed (1). While SRLVs have been widely documented in sheep and goats, almost in all continents (16), the infection in wild ruminants is still poorly investigated and understood (17).

Studies investigating the circulation of SRLV in wild ruminants, although limited, have reported both serological and molecular evidence of infection. Antibodies were detected in Rocky Mountain goats (*Oreamnos americanus*) in North America (2004–2008) (18) and in one mouflon (*Ovis aries musimon*) in Slovenia (2017–2018) (19). In addition, SRLV antibodies have been detected in red deer, roe deer, and mouflons in Spain (20), whereas no seropositivity was observed in mouflon, mule deer (*Odocoileus hemionus ssp.*), chamois (*Rupicapra rupicapra*), red deer and roe deer, in the EU (Italy, Spain and Germany) and USA (21–25).

First reports of molecular detection by nested end-point PCR of SRLV genotype B in wild ruminants were published in 2008, describing SRLV-positive wild ibexes, members of the *Bovidae* family, identified from samples collected in previous years during surveillance activities in the French Alps (26). Recently, in 2024, SRLV positivity was reported in two red deer (*Cervidae* family) from Poland, using a nested real-time PCR assay (17). Proviral DNA detection was also reported by Guiguen et al. (27) who identified SRLV in peripheral blood mononuclear cells from mouflon–sheep hybrids experimentally infected with a CAEV strain using a semi-quantitative PCR.

Among cervids, roe deer are widespread in Italy’s Alps and Apennines, which spans the peninsula from north to south. Following a historical population decline that affected much of Europe and brought the species close to extinction, roe deer populations have recovered and are now broadly distributed across forested and semi-natural habitats throughout the continent. This species has managed to adapt to an environment which is increasingly altered by human activities (28, 29). In Italy, the species is primarily distributed across the Alpine and Apennine regions, although currently expanded into lowland, agricultural, and peri-urban environments, with smaller and more fragmented populations of subspecies *C. c. italicus* still present in southern areas of the peninsula (30). The increasing density and spatial expansion of roe deer populations have enhanced interactions among wildlife, domestic animals, and humans. Given the extensive circulation of small ruminant lentiviruses (SRLV) in sheep and goats in central Italy, specifically Umbria and Marche regions (13–15) and the widespread presence of pasture-based farming systems, that facilitate contact between domestic ruminants and wildlife (31–33), a pathogen monitoring program on roe deer (*Capreolus capreolus*) was implemented in these regions between 2023 and 2025.

This study aimed to investigate the circulation of SRLV at the wildlife–livestock interface by focusing on a wild ruminant species inhabiting an area characterized by a high density of domestic small ruminants and endemic SRLV infection. Our findings contribute to shed light on the potential role of wildlife in the SRLV epidemiological dynamics.

## 2 Method

### 2.1 Sample collection and processing

The monitoring program on SRLV in roe deer took place between February 2023 and December 2025 and was based on testing carcasses primarily submitted by wildlife rescue centers located in Umbria and Marche regions. In addition, roe deer carcasses from road accidents were also included. All samples derived from animals found dead or deceased at wildlife rescue centers. Necropsy was performed on each carcass submitted to Istituto Zooprofilattico Sperimentale dell’Umbria e delle Marche (IZSUM) local laboratories to assess the underlying cause of death and monitor the health status of the area, considering the ecological sentinel function performed by this wild species. For SRLV detection, spleen was collected as the primary diagnostic organ from each animal, from February 2023 onward. Starting in May 2024, lung tissue and blood clots were introduced as alternative or complementary matrices to spleen samples. To maximize diagnostic sensitivity when primary tissues were degraded, insufficient, or unavailable, these matrices were pooled (comprising spleen and/or lung and/or blood clot). However, due to the advanced decomposition of some specimens, collecting a complete set of samples from every animal was not always feasible. Notably, in cases where carcasses had been heavily compromised by predators, target organs, including the spleen itself, were frequently missing or entirely unavailable for sampling. All samples were analyzed at the National Reference Laboratory for Ruminant Retroviruses (CEREL) at IZSUM.

### 2.2 SRLV molecular detection and characterization

After the samples were homogenized, viral RNA extraction was performed manually according to the manufacturer’s instructions, using the QIAamp Viral RNA Mini Kit-Spin Protocol (Qiagen, Hilden, Germany). The amplification protocol from RNA included an initial reverse transcription phase, followed by a nested PCR targeting the *gag-pol* region of about 900 bp, adapted from previous works (13, 32). This fragment comprises 822 nt of 3′ end of the *gag* gene and 102 nt of the 5′ end of the *pol* gene. The Icelandic 1,514 strain was used as a positive control for genotype A (Genbank accession number M10608), and CAEV-Co for genotype B (Genbank accession number M33677). PCR results were analyzed via electrophoresis on a 2% agarose gel. For genetic characterization, Sanger sequencing was attempted using the *gag-pol* PCR amplicon, while for further characterization a primer walking approach was developed for the entire *gag* gene (about 1,500 bp), which allows the amplification of three overlapping fragments, the first one of about 300 nt, the second of about 600 nt, and the third of about 800 nt corresponding to the gene fragment amplified by the diagnostic PCR (Supplementary material). Following the nested *gag-pol* PCR and the PCR of the whole *gag* gene, bands of the expected size were excised from agarose gel and purified using the QIAquick Gel Extraction kit (Qiagen, Hilden, Germany) following the manufacturer’s protocol. The purification product was used as a template in the sequencing reaction conducted using the BrilliantDye™ Terminator Cycle Sequencing v3.1 Kit (NimaGen BV, Nijmegen, The Netherlands). The sequencing reaction products were subsequently precipitated using the DyeEx 2.0 Spin Kit (Qiagen, Hilden, Germany) following the manufacturer’s protocol. Following denaturation with formamide, the products were loaded into the ABI PRISM 3130 Genetic Analyzer automatic sequencer (Applied Biosystems, Foster City, CA, USA). The forward and reverse sequences of each sample were analyzed and assembled using Geneious software v.2025.1.3 (34), yielding the consensus sequence, which was then compared and aligned with other reference sequences downloaded from GenBank using ClustalX 2.1 (35) and utilized for genotyping. The aligned sequences were further refined and trimmed using BioEdit v.7.2.5 (36), Seqscape v3.0 (ThermoFisher, Waltham, Massachusetts, USA), MEGA X (37) and FinchTV v1.4.0 (Geospiza, USA). The obtained *gag-pol* sequence of 832 bp was submitted to Genbank under the accession number PX724596.

To characterize the new sequence, a phylogenetic analysis was performed, including related sequences retrieved from NCBI through a BLAST search (38), together with genotype A references. The final alignment was 941 bp long and comprised 232 genotype A sequences and 19 genotype B sequences, which were included as an outgroup. The phylogenetic analysis was performed using the maximum likelihood (ML) method implemented in IQ-TREE v3.0.1 (39) under the best-fitting model (GTR + F + R5) (40), and branch support was assessed using 100 non-parametric bootstrap replicates. The tree was visualised and collapsed where necessary, using Figtree 1.4.4 (41).

## 3 Results

Between 1st January 2023 and 31st December 2025, a total of 179 roe deer were sampled in central Italy, Umbria and Marche regions, collecting 245 samples. Most animals (n = 161) were sampled in Marche and the remaining 18 roe deer were sampled in Umbria. The highest number of samples were received in 2024 (107/245 samples obtained from 83 roe deer, which amounts to 43.67%) and 2025 (95/245 samples obtained from 53 roe deer, which amounts to 38.77%) (Figure 1). This sampling effort was successful in covering both Umbria provinces (Perugia and Terni) from 2023 to 2024 and four out of the five provinces in Marche (Ancona, Ascoli-Piceno, Fermo, and Macerata) (Figure 1). The 245 samples included 132 spleens (53.87%), 56 lungs (22.85%), 43 organ pools (17.55%) and 14 blood clots (5.71%). During 2023, collaborative relationships were strengthened with the CRAS (Wildlife Rescue Center), which retrieves carcasses and delivers them to IZSUM laboratories, and the procedures for delivery were better defined. This has led to a gradual increase in the number of specimens delivered. One roe deer spleen tested positive for SRLV by PCR and was identified in Marche region (Fermo province) in 2025. At the time of the necropsy, the animal was identified as an adult female presenting macroscopic lesions consistent with traumatic events, including multiple displaced fractures, abdominal hemorrhagic effusion, and diaphragmatic hernia, indicating death due to trauma most probably by road accident.

**Figure 1:**
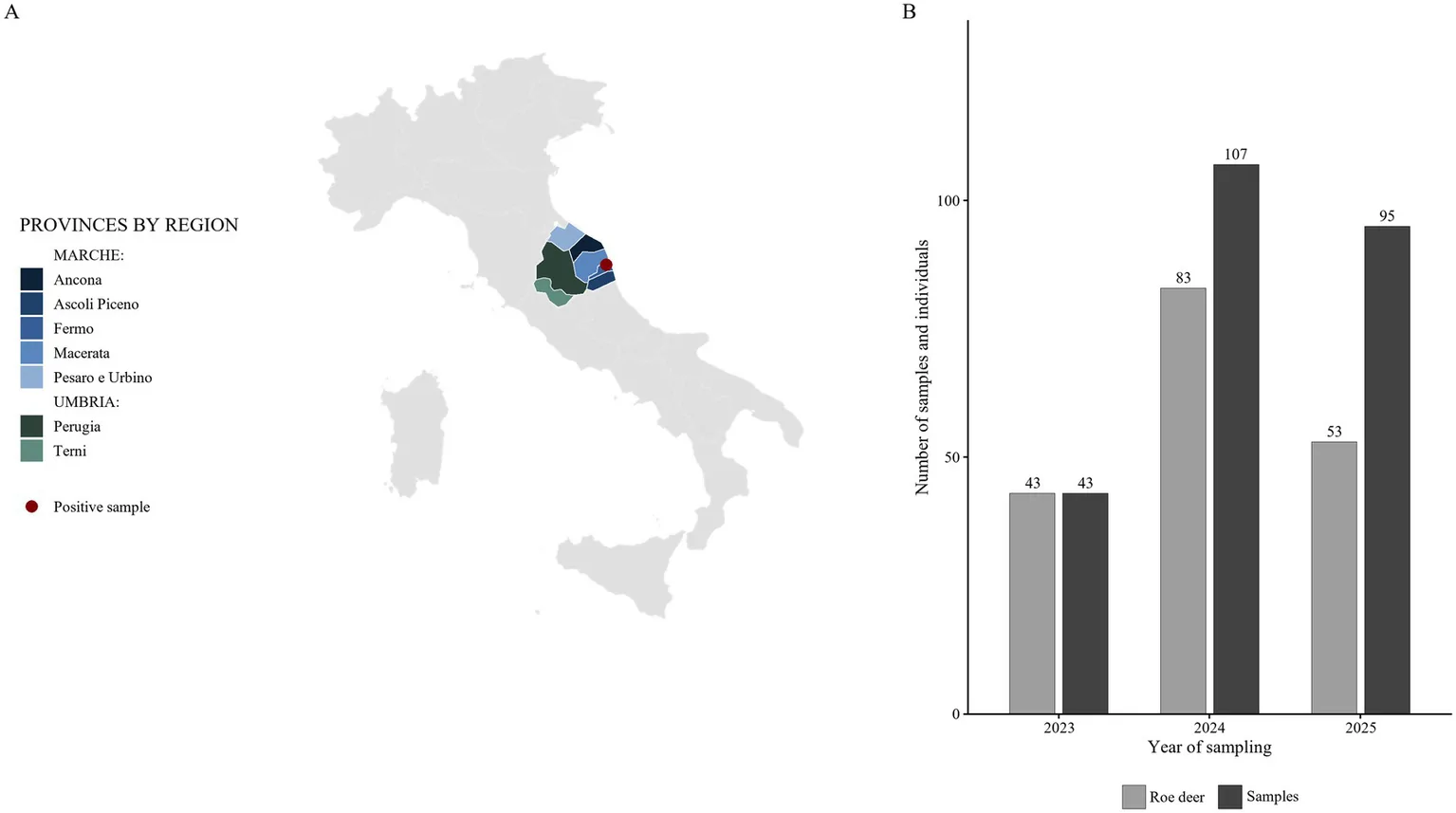
A. Map showing the origin of the positive sample and the region and provinces of sampled animals. B. Samples assessed per individual and year.

Sanger sequencing of the *gag-pol* region yielded an 832 bp sequence. Attempts in whole *gag* gene Sanger sequencing produced consistent results for one out of three segments. The two sequencing protocols yielded consistent findings, validating the robustness of the methods used. The phylogenetic analysis showed the SRLV from the roe deer belonged to the A23 sub-genotype, sporadically identified in domestic wild ruminants in Italy (13, 15). The characterized SRLV (PX724596_deer/Italy/500_MA/2025) clustered within a well-supported group including other Italian SRLVs from domestic small ruminants (Figure 2) identified in Southern Italy. This genetic group was composed of two well-supported genetic sub-groups. In detail one sub-group was composed by the roe deer SRLV and one SRLV identified from sheep in Sicily in 2012 (LR723582_sheep/Italy/134_SI/2012). The other was composed by three SRLVs, of which one was identified in Sicily from goat in 2012 (LR723579_goat/Italy/111_SI/2012) and two from sheep in a neighboring region in 2016 (Calabria region, LR732257_sheep/Italy/223_CA/2016 and LR732258_sheep/Italy/224_CA/2016) (Figure 2).

**Figure 2:**
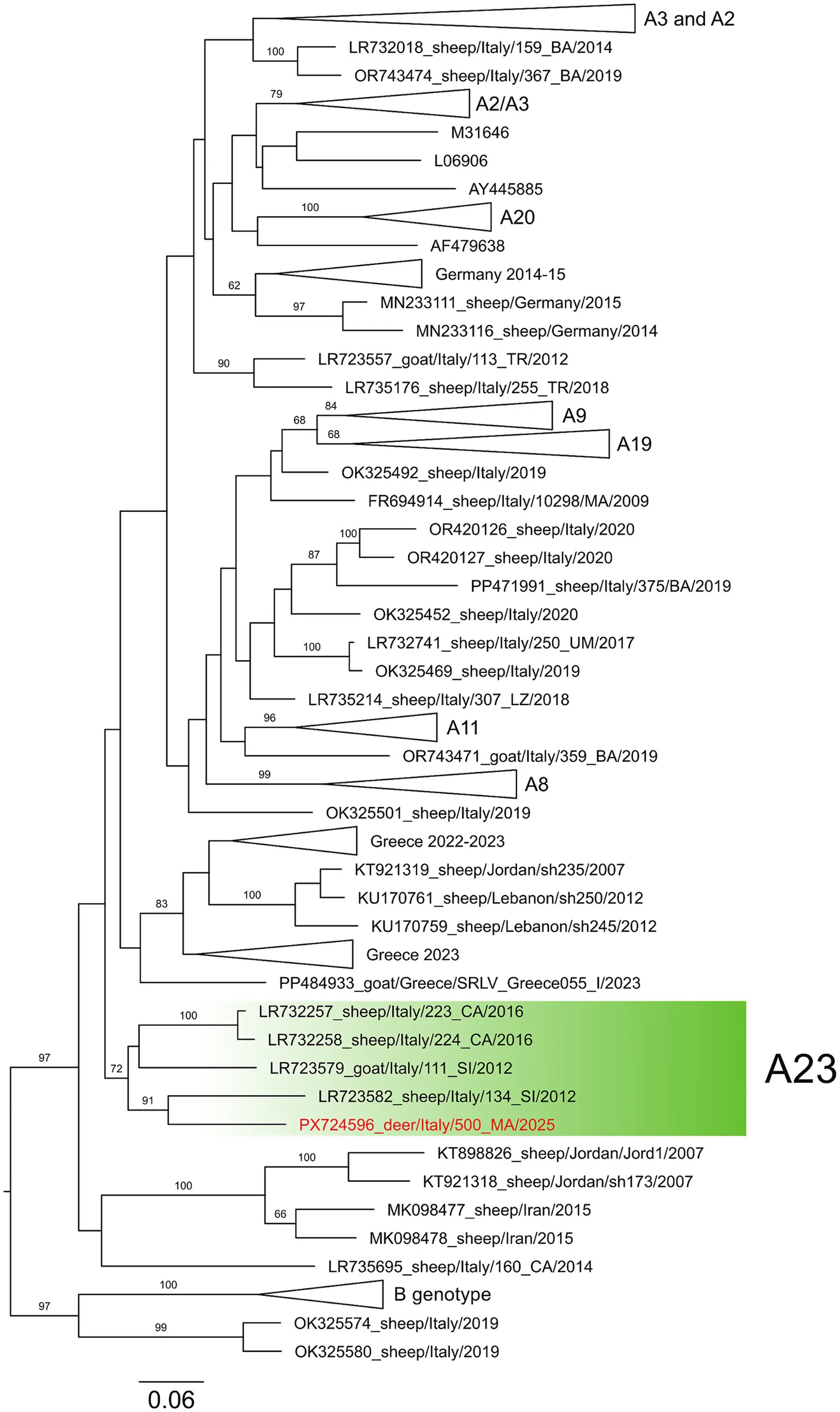
Phylogenetic tree of the *gag-pol* region (941 bp). Bootstrap values ≥60 obtained from 100 non-parametric bootstrap replicates are reported. The SRLV identified in this study is indicated in red, and the clade in which it clusters is shaded in green. Clades with bootstrap values ≥60 and containing more than three sequences were collapsed.

## 4 Discussion

The wild ruminant monitoring program conducted in two regions of Central Italy between 2023 and 2025 led to the first detection of an SRLV strain in a roe deer. To the best of our knowledge, this represents the first molecular report of SRLV in roe deer species worldwide and the second detection of SRLV in the *Cervidae* family in Europe. The roe deer in question was reported as an adult female animal rescued in Marche region and most probably died as a result of road-accident-related trauma. The characterized SRLV belonged to the A genotype, A23 sub-genotype, genetically related to SRLV from sheep that circulated in Southern Italy between 2012 and 2016. This finding suggests that habitat sharing with extensive farming and subsequent wildlife movement across the Apennines may represent a key factor for SRLV infection in wild and domestic ruminants. Another hypothesis is that the grouping of strains from Southern Italy may not necessarily indicate a direct epidemiological link, but rather a genetic correlation consistent with the restricted availability of homologous sequences. The identification of SRLV in a roe deer provides the first molecular evidence of natural infection in this species. While previous studies in Europe have primarily focused on red deer, our findings expand the known host range of SRLV to include one of the most widespread and ecologically successful European cervids. Being only the second report of SRLV in the *Cervidae* family within Europe, this discovery raises the possibility that interspecies transmission may occur more frequently than previously documented. However, additional evidence is necessary, particularly from regions where high-density wild populations overlap with infected domestic flocks. SRLVs have been traditionally regarded as pathogens strictly associated with the *Bovidae* family, specifically adapted to domestic sheep and goats. However, the detection of the virus in the distinct taxonomic family of *Cervidae* highlights the ability of SRLV to occur beyond its main domestic hosts. This inter-family spillover indicates that the molecular mechanisms of viral entry and replication are sufficiently conserved to bypass the phylogenetic distance between bovids and cervids. Such host-range expansion raises critical questions regarding the role of cervids as possible alternative reservoirs or ‘dead-end hosts,’ and emphasizes the need for a multi-species approach in SRLV surveillance programs. The low frequency of positive cases reported so far in wildlife does not necessarily indicate negligible epidemiological relevance, but rather reflects the challenges associated with detecting lentiviral infections characterized by low viral loads, lifelong persistence, and often asymptomatic courses. The identification of a SRLV from a road-accident roe deer led to the conclusion that as most wildlife carcasses are recovered after vehicle collisions, excluding these individuals from surveillance based on overt trauma may lead to a significant underestimation of pathogen prevalence. Systematic screening for infectious agents should therefore be performed regardless of the apparent cause of death to ensure a comprehensive assessment of population health. The present case report adds to the limited body of molecular data on SRLV infection in wildlife and aligns with recent findings, suggesting that SRLV traditionally associated with domestic small ruminants may sporadically infect cervids under natural conditions. Although SRLVs have been extensively studied in domestic sheep and goats, their occurrence in wild ruminants remains poorly documented. Most investigations conducted in wildlife have relied on serological approaches and have generally reported negative or sporadic positive results (18, 19, 21–25). Only a limited number of studies provided molecular evidence of SRLV infection in wild ruminants, highlighting the rarity of confirmed cases despite extensive sampling efforts. In this framework, the recent study in Poland represents a key reference, as it demonstrated, for the first time, the presence of SRLV proviral DNA in free-ranging cervids using molecular techniques (17). Specifically, the detection of SRLV sequences closely related to CAEV in red deer suggests that natural exposure and infection of cervids by SRLVs may occur. The findings reported in the present case are consistent with and expand upon this emerging evidence. Continued surveillance, coupled with molecular characterization of detected strains, will be essential to clarify the epidemiological significance of these findings, including the potential role of wild ruminants in the maintenance or spillover of SRLVs. An additional aspect that should be considered when interpreting the limited availability of epidemiological data on roe deer concerns hunting regulations and access to biological samples. In Italy, roe deer hunting is exclusively based on selective culling, which is regulated by strict temporal, spatial, and demographic criteria. While this management framework is effective for population control, it limits the possibility of carrying out health monitoring out of the hunting season and/or in protected areas. Consequently, access to representative samples from free-ranging roe deer for pathogen surveillance is frequently constrained. In this context, animals admitted to wildlife rescue and rehabilitation centers due to trauma, weakness, or simply road-killed represent an important, albeit non-random, source of biological samples of species and age classes that are otherwise difficult to sample. The inclusion of samples obtained through these underscored networks enhances the sensitivity of monitoring programs and increases the likelihood of detecting sporadic or unexpected infections, such as the SRLV-positive case described in the present report. This integrated approach, combining selective hunting-based surveillance with data derived from wildlife rescue centers, may therefore represent an effective strategy to improve pathogen detection in roe deer populations and to better characterize the epidemiological landscape of infectious agents circulating in wild ruminants. In conclusion, this case underscores the importance of maintaining and expanding wildlife health monitoring programs to detect unexpected infections in free-ranging species. Even sporadic detections, when integrated into robust surveillance framework and supported by comparable studies, offer valuable insights into pathogen ecology and host range. Such findings contribute to a more comprehensive understanding of infectious disease dynamics at the wildlife–livestock interface.

## Data Availability

All the data used in this study are generated from our experiments and available in the manuscript.
