# Supplementary material for: First molecular detection of small ruminant lentivirus in a roe deer (Capreolus capreolus) in Italy
Source: Front Vet Sci. 2026 Oct 9;13:1900987. doi: 10.3389/fvets.2026.1900987 (PMC13692785; doi:10.3389/fvets.2026.1900987)
Supplement: Supplementary file 1 [file Table_1.DOCX]

Supplementary Material

**Molecular Characterization of Small Ruminant Lentiviruses (SRLVs)**

1. **Diagnosis of SRLV**
   1.1. **RT-PCR of *gag-pol* region**

RNA was extracted manually according to the manufacturer’s instructions, using the QIAamp Viral RNA Mini Kit (Qiagen, Hilden, Germany). The PCR protocol is based on the amplification of about 800 bp *gag-pol* region, following the modified protocol of Grego et al. The list and sequence of primers is available in Table 1.

**Table 1. Primers used for the diagnosis of SRLV by RT-PCR from Grego et al 2007.**

| Target | Primer name | Sequence |
| --- | --- | --- |
| *gag-pol* | GAG-F1 | 5’-TGGTGARKCTAGMTAGAGACATGG-3’ |
|  | POL-R1 | 5’-CATAGGRGGHGCGGACGGCASCA-3’ |
|  | GAG-F2 | 5’-CAAACWGTRGCAATGCAGCATGG-3’ |
|  | POL-R2 | 5’-GCGGACGGCASCACACG-3’ |

F: forward

R: reverse

The reverse transcriptase mix was carried out in 20 μl reaction volume consisting of the following reaction mix:

**Retrotranscription (RT) phase**

| **Reagent** | **Initial concentration** | **µl/reaction** |
| --- | --- | --- |
| H_2_O + DEPC |  | 8,25 |
| Strand Buffer | 5x | 4 |
| dNTPs | 10 mM | 1 |
| Random primers | 50 µM | 1 |
| RNAse Inhibitor | 40 U/µl | 0,25 |
| SuperScript® III | 200 U/µl | 0,5 |
| **Mix Volume** |  | **15** |
| **RNA** |  | **5** |
| **Final Volume** |  | **20** |

- SuperScript® III Reverse Transcriptase (Invitrogen) or equivalent.

**Thermal profile**

| **Cycle** | **Temperature** | **Time** |
| --- | --- | --- |
| 1 | 37°C | 90 min |
| 1 | 65°C | 10 min |

**I *gag-pol* PCR**

| **Reagent** | **Initial Concentration** | **µl/reaction** |
| --- | --- | --- |
| Buffer | 10x | 5 |
| MgCl_2_ | 50 mM | 2 |
| dNTPs | 10 mM | 1 |
| Primer F – GAGF1 | 20 µM | 1,5 |
| Primer R – POLR1 | 20 µM | 1,5 |
| BSA | 10x | 5 |
| Taq Platinum™ | 5 U/µl | 0,2 |
| H_2_O + DEPC | - | 30,8 |
| **Mix volume** |  | **47** |
| **cDNA** |  | **3** |

- Platinum Taq DNA Polymerase (Invitrogen) or equivalent.

**Thermal profile**

| **Cycle** | **Temperature** | **Time** |
| --- | --- | --- |
| 1 | 94°C | 2 min |
| 40 | 94°C | 45 sec |
|  | 55°C | 1 min |
|  | 72°C | 1 min |
| 1 | 72°C | 10 min |

**nested *gag-pol* PCR**

| **Reagent** | **Initial Concentration** | **µl/reaction** |
| --- | --- | --- |
| Buffer | 10x | 5 |
| MgCl_2_ | 50 mM | 2 |
| dNTPs | 10 mM | 1 |
| Primer F – GAGF2 | 20 µM | 1,5 |
| Primer R – POLR2 | 20 µM | 1,5 |
| BSA | 10x | 5 |
| Taq Platinum™ | 5 U/µl | 0,2 |
| H_2_O + DEPC |  | 30,8 |
| **Mix volume** |  | **47** |
| **I *gag-pol* PCR template** |  | **3** |

**Thermal profile**

| **Cycle** | **Temperature** | **Time** |
| --- | --- | --- |
| 1 | 94°C | 2 min |
| 40 | 94°C | 45 sec |
|  | 65°C | 1 min |
|  | 72°C | 1 min |
| 1 | 72°C | 10 min |

**Target length: about 800 bp**

1.2. **Sanger sequence protocol of the *gag-pol* region**

Positive samples were subjected to Sanger sequencing. The I *gag-pol* PCR template was purified using the following products, depending on the quality of the band.

Reagents:

- QIAquick PCR Purification Kit (Qiagen, Hilden, Germany) or equivalent.

- ExS-Pure™ Enzymatic PCR Purification Kit (NimaGen, Nijmegen, The Netherlands) or equivalent.

Two replicates of the purified product were set up for each sample using the forward primer (GAG-F2) and two replicates using the reverse primer (POL-R2). Both GAG-F2 and POL-R2 were used at a concentration of 3.2 μM (Tables 2-3).

**Table 2. Reaction mix related to GAG-F2 primer.**

| **Reagent** | **Volume** |
| --- | --- |
| BrilliantDye v 3.1 RR premix | 1 |
| 5X sequencing Buffer | 1.5 |
| GAG-F2 primer 3.2 µM | 1 |
| DNA* | - |
| H_2_O** | - |
| Total mix | 20 |

**Table 3 Reaction mix related to POL-R2 primer.**

| **Reagent** | **Volume** |
| --- | --- |
| BrilliantDye v 3.1 RR premix | 1 |
| 5X sequencing Buffer | 1.5 |
| POL-R2 primer 3.2 µM | 1 |
| DNA* | - |
| H_2_O** | - |
| Total mix | 20 |

*: the DNA amount depends on the quality of the sample.

**: H2O amount is calculated to reach the final volume of 20 µl.

- BrilliantDye™ Terminator v3.1 Cycle Sequencing Kit 100 rxn (NimaGen, Nijmegen, The Netherlands) or equivalent.

The prepared reaction mixes were incubated according to the following thermal profile: Taq polymerase activation at 96 °C for 45 s, followed by 25 cycles with denaturation at 96 °C for 10 s, annealing at 50 °C for 5 s and extension at 60 °C for 4 min. The sequencing reaction products were subsequently precipitated using the DyeEx 2.0 Spin Kit (Qiagen, Hilden, Germany) following the manufacturer’s protocol and denatured at 95 °C for 5 min after the addition of HiDi Formamide (Applied Biosystems Life Technologies, Warrington, United Kingdom). Following denaturation, the products were loaded into the ABI PRISM 3130 Genetic Analyzer automatic sequencer (Applied Biosystems, Foster City, CA, United States).

2. **Experimental protocol for the characterization of the complete *gag* gene of SRLV**
   2.1. **Primer walking procedure**

The DNA and RNA extraction phase is analogue to that of the *gag-pol* protocol. The genomic region corresponding to the complete *gag* gene (reference accession number M10608, position from 340 nt to 1818 nt) was divided into three fragments, represented by three different PCR protocols. The list and sequence of primers is available in Table 4.

**Table 4. Primers used for the experimental whole gag sequencing protocol.**

| *gag* gene fragment | Primer Name | Sequence | Source |
| --- | --- | --- | --- |
| 1 | MVV F0 | 5’-AAGTAAGGTAAGAGAGACACCTACTGG-3’ | [4] |
|  | NestSRLV R3 | 5’-CCTTCTGTCAAGGTCTCCTTCCC-3’ | [5] |
| 2* | MVV F2 | 5’-GACAGAAGGGAACTGTCTATGGGC-3’ | [4] |
|  | 972deg rev | 5’-TGCCTGATCCATRTTRGCTTG-3’ | [3] |
|  | MVV F2 | 5’-GACAGAAGGGAACTGTCTATGGGC-3’ | [4] |
|  | Gag2 rev | 5’-TGTGCTCTATTCCCAGGCATCAT-3’ | [6] |
| 3 | GagF2 | 5’-CAAACWGTRGCAATGCAGCATGG-3’ | [1] |
|  | MVVR0 | 5’-GGTGGTGCTTCTGTTACAACATAGG-3’ | [4] |

*: Two sets of primer are present for the second fragment since it covers a variable region within gag gene, so an alternative set was found.

To maximize PCRs yield, the Platinum™ SuperFi™ DNA Polymerase and the AmpliTaq Gold™ DNA Polymerase were selected (ThermoFisher Scientific, Waltham, MA, USA). Samples were tested from DNA, and in parallel from cDNA obtained with reverse transcription phase, as in the *gag-pol* detection.

The PCR reaction mixes of the first, second and third fragments are reported in Tables 5-6a-6b-7 respectively.

**Table 5. Reaction mix for the first *gag* fragment.**

| **Reagent** | **Initial concentration** | **Volume** |
| --- | --- | --- |
| Platinum SuperFi PCR MasterMix | 2X | 25 |
| Primer MVV F0 | 10 µM | 2.5 |
| Primer NestSRLV R3 | 10 µM | 2.5 |
| SuperFi GC Enhancer | 5X | 10 |
| H2O+DEPC | - | 5 |
| **Mix volume** |  | **45** |
| **cDNA** |  | **5** |
| **Total Volume** |  | **50** |

**Table 6a. Reaction mix for the second *gag* fragment.**

| **Reagent** | **Initial concentration** | **Volume** |
| --- | --- | --- |
| Platinum SuperFi PCR MasterMix | 2X | 25 |
| Primer MVV F2 | 10 µM | 2.5 |
| Primer 972deg rev | 10 µM | 2.5 |
| SuperFi GC Enhancer | 5X | 10 |
| H2O+DEPC | - | 5 |
| **Mix volume** |  | **45** |
| **cDNA** |  | **5** |
| **Total Volume** |  | **50** |

**Table 6b. Alternative reaction mix for the second *gag* fragment.**

| **Reagent** | **Initial concentration** | **Volume** |
| --- | --- | --- |
| Buffer | 10X | 5 |
| MgCl_2_ | 25 mM | 4 |
| dNTPs | 10 mM | 1 |
| Primer MVV F2 | 10 µM | 1.25 |
| Primer Gag2 rev | 10 µM | 1.25 |
| AmpliTaq Gold DNA | 5 U/µl | 0.25 |
| H2O+DEPC | - | 5 |
| **Mix volume** |  | **45** |
| **cDNA** |  | **5** |
| **Total Volume** |  | **50** |

**Table 7. Reaction mix for the third *gag* fragment.**

| **Reagent** | **Initial concentration** | **Volume** |
| --- | --- | --- |
| Platinum SuperFi PCR MasterMix | 2X | 25 |
| Primer GAG F2 | 10 µM | 2,5 |
| Primer MVV R0 | 10 µM | 2,5 |
| SuperFi GC Enhancer | 5X | 10 |
| H2O+DEPC | - | 5 |
| **Mix volume** |  | **45** |
| **cDNA** |  | **5** |
| **Total Volume** |  | **50** |

The thermal profiles are reported in the following tables:

| **Cycle** | **Temperature** | **Time** |
| --- | --- | --- |
| 1 | 98°C | 30 sec |
| 45 | 98°C | 10 sec |
|  | 62°C (first fragment)  58°C (second fragment)  57°C (third fragment) | 45 sec |
|  | 72°C | 1 min |
| 1 | 72°C | 5 min |

| **Cycle** | **Temperature** | **Time** |
| --- | --- | --- |
| 1 | 95°C | 10 min |
| 42 | 95°C | 1 min |
|  | 62°C (alternative second fragment) | 1 min |
|  | 72°C | 1 min |
| 1 | 72°C | 5 min |

PCR templates were read through classic electrophoresis on 2% agarose gel, using Midori Green Advance (NIPPON Genetics Europe GmbH, Düren, Germany).

2.2. **Sequencing procedure of the *gag* gene fragments**

Purification of PCR products was done using QIAquick PCR Purification Kit (Qiagen, Hilden, Germany) following the manufacturer's protocol. The purification product was used as a template in the sequencing reaction (chain termination PCR) conducted using the BrilliantDyeTM Terminator Cycle Sequencing v3.1 Kit (NimaGen BV, Nijmegen, The Netherlands). Two replicates of the sequencing reaction were set up for each sample using the forward primer and two replicates using the reverse primer. Both primers were used at a concentration of 3.2 µM. The prepared reaction mixes were loaded into the thermocycler and incubated according to the following thermal profile: Taq polymerase activation at 96°C for 45 s, followed by 25 cycles with denaturation at 96°C for 10 s, annealing at 50°C for 5 s and extension at 60°C for 4 min. The sequencing reaction products were subsequently precipitated using the Optima DTR 8-Well Strip Kit (EdgeBio, San Jose, CA, USA) following the manufacturer's protocol and denatured at 95°C for 5 minutes after the addition of HiDi Formamide (Applied Biosystems Life Technologies, Warrington, UK). Following denaturation, the products were loaded into the SeqStudio™ 8 Flex Genetic Analyzer automatic sequencer (Applied Biosystems, Foster City, CA, USA).

***References***

1. Grego E, Bertolotti L, Quasso A, Profiti M, Lacerenza D, Muz D, Rosati S. Genetic characterization of small ruminant lentivirus in Italian mixed flocks: evidence for a novel genotype circulating in a local goat population. J Gen Virol. 2007 Dec;88(Pt 12):3423-3427. doi: 10.1099/vir.0.83292-0. PMID: 18024912.
2. Gobbi P, Tassoni L, Tinelli E, Scoccia E, Mrabet S, Orrico M, Righi C, Maresca C, D'Avino N, Passamonti F, Giammarioli M, Feliziani F, Beato MS. Genetic landscape of Italian SRLV: insights from passive surveillance-based phylogenetic analysis. Front Microbiol. 2026 Jan 9;16:1745687. doi: 10.3389/fmicb.2025.1745687. PMID: 41586368; PMCID: PMC12827567.
3. Gobbi P. Molecular surveillance strategies for Small Ruminant Lentiviruses: A case study from Italy. PhD thesis.
4. L'Homme Y, Ouardani M, Lévesque V, Bertoni G, Simard C, Pisoni G. Molecular characterization and phylogenetic analysis of small ruminant lentiviruses isolated from Canadian sheep and goats. Virol J. 2011 Jun 3;8:271. doi: 10.1186/1743-422X-8-271. PMID: 21639904; PMCID: PMC3123287.
5. Schaer, J.; Cvetnic, Z.; Sukalic, T.; Dörig, S.; Grisiger, M.; Iscaro, C.; Feliziani, F.; Pfeifer, F.; Origgi, F.; Zanoni, R.G.; et al. Evaluation of Serological Methods and a New Real-Time Nested PCR for Small Ruminant Lentiviruses. Pathogens 2022, 11, doi:10.3390/pathogens11020129.
6. Laamanen, I.; Jakava-Viljanen, M.; Sihvonen, L. Genetic Characterization of Maedi-Visna Virus (MVV) Detected in Finland. Vet Microbiol 2007, 122, 357–365, doi:10.1016/j.vetmic.2007.02.002.
